# Editorial for the Special Issue “Environmental Exposure and Reproductive Health”

**DOI:** 10.3390/toxics12030216

**Published:** 2024-03-14

**Authors:** Pan Yang

**Affiliations:** Department of Public Health and Preventive Medicine, School of Medicine, Jinan University, Guangzhou 510632, China; yangpan@jnu.edu.cn

## 1. Introduction

In recent decades, the decline in human fertility has emerged as a significant public health concern, garnering global attention. There are numerous factors contributing to the decline in fertility, with environmental pollution being notable [1,2]. Toppari et al. proposed the “endocrine disrupting hypothesis”, suggesting that exposure to endocrine-disrupting chemicals (EDCs) during fetal or infant development may affect the development of reproductive organs and subsequently have adverse effects on reproductive health in adulthood [3]. With the rapid development of industrialization and urbanization, environmental contaminants are ubiquitous in the global environment. For example, multiple EDCs have been detected in indoor dust, surface water, soil, and other environmental media [4,5,6]. Consequently, humans are exposed to numerous EDCs through ingestion, inhalation, and dermal contact, which can adversely affect their reproductive health through various pathways. Further research is needed to explore the long-term effects of EDC exposure on human fertility and to develop strategies to minimize exposure levels.

The Special Issue “Environmental Exposure and Reproductive Health” has gathered and published 10 contributions that focus on the reproductive effects of environmental chemicals, particularly their impact on germ cells, reproductive organs, and reproductive outcomes. Three studies have assessed the impact of environmental pollutants on germ cells.

## 2. An Overview of Published Articles

Feng’s article (contribution 1) investigated the toxic effects of bisphenol AF (BPAF) on the porcine Sertoli cell line (ST cells), essential for testis development and spermatogenesis. They found that BPAF exposure induces apoptosis and cell cycle arrest in ST cells by activating ROS-mediated pathways. The authors concluded that BPAF impairs the male reproductive system and suggested further toxicological assessments. These findings provide a foundation for future toxicological assessments. Mourike’s article (contribution 2) delved into the differential impact of imidacloprid and its bioactive metabolite desnitro-imidacloprid on antral follicle growth and steroidogenesis. Utilizing an in vitro model of antral follicles from mice, the study uncovered distinct effects of imidacloprid and desnitro-imidacloprid on follicle growth, steroid hormone levels, and gene expression patterns, suggesting nuanced mechanisms of ovarian toxicity associated with neonicotinoids. Furthermore, Mathur’s article (contribution 3) evaluated the toxic effects of hospital effluent on sperm quality. The study showed the impact of treated hospital on sperm quality in mice. Employing morphological and geometric morphometric analyses of sperm, the research revealed significant abnormalities in sperm morphology and head dimensions due to exposure to treated effluent, underscoring that the presence of toxicants in such effluents could potentially affect sperm quality. 

Exposure to heavy metals, organic pollutants, and other environmental contaminants during pregnancy can potentially pose risks to the health of both the fetus and the mother. Four papers in this Special Issue evaluate the effects of environmental pollutants on pregnant women. Yang’s article (contribution 4) employed various regression models to explore the impact of co-exposure to polycyclic aromatic hydrocarbons (PAHs) and phthalates (PAEs) on blood-cell-based inflammatory indicators in early pregnant women. They found that increased inflammatory indicators during the first trimester were linked to co-exposure to PAHs and PAEs, among which mono-octyl phthalate might be the major contributor. Dai and co-authors’ article (contribution 5) focused on early pregnant women and their urinary PAH metabolites, aiming to establish connections with liver function. Employing multiple linear regression and Bayesian kernel machine regression models, the results showed associations between specific urinary PAH metabolites and altered liver function parameters. Notably, increments in certain PAH metabolites correlated with elevated alanine aminotransferase, aspartate aminotransferase, and total bile acid levels, underscoring the potential adverse impact of PAH exposure on liver function during early pregnancy. Another study by Akgöl (contribution 6) examined the relationship between spontaneous abortion and blood pesticide and polychlorinated biphenyl (PCB) levels. This study surveyed 200 patients, segregating them into two groups: cases with spontaneous abortion and those with normal deliveries. Employing advanced mass spectrometry techniques, a diverse range of pesticides and PCBs were quantitatively analyzed in collected blood samples. The investigation revealed higher levels of specific chemicals, including β-hexachlorocyclohexane, delta-hexachlorocyclohexane, hexachlorobenzene, and various PCBs, in the spontaneous abortion group compared to the control. The identification of a high exposure to organochlorine compounds underscored the potential role of these chemicals in spontaneous abortions, signifying the necessity for further exploration and regulatory attention toward minimizing such exposures. Moreover, Zhang’s article (contribution 1) investigated exposure to heavy metals in patients with unexplained recurrent miscarriage, employing inductively coupled plasma–mass spectrometry to measure metal levels in serum samples. Noteworthy findings highlighted negative associations of calcium and selenium exposure with miscarriage, while lead exposure demonstrated a positive correlation. After adjusting for relevant factors, these outcomes suggested potential links between specific metal elements in the blood and the risk of unexplained recurrent miscarriage. In addition, environmental exposure can also impact the development of reproductive organs in offspring. Lahimer’s article (contribution 8) utilized histological and immunohistological analyses to evaluate the effects of maternal exposure to chlorpyrifos (CPF) and/or a high-fat diet (HFD) on the reproductive organs of rat offspring. Remarkably, the levels of Kisspeptin and GnRH receptor in the testis of rat offspring declined in the CPF group and HFD group, respectively, indicating that the offspring’s testis is more sensitive to obesity and pesticide exposure than the offspring’s ovary.

Environmental exposure is a major health problem worldwide, making it crucial to explore effective strategies for mitigating the adverse impacts on the reproductive system. A study by Sevim’s (contribution 9) identified probiotic mitigation of neonicotinoid toxicity, highlighting the potential protective effects of the probiotic *Saccharomyces boulardii* against neonicotinoid-induced reproductive toxicity in male rats. This study observed degeneration and necrosis of spermatocytes and increased oxidative stress and apoptosis markers due to acetamiprid and imidacloprid exposure. The supplementation with *Saccharomyces boulardii* mitigated these toxic effects, indicating a potential intervention strategy against pesticide-induced reproductive toxicity. Research suggests that per- and polyfluoroalkyl substance (PFAS) exposure may have adverse effects on human health, including reproductive function. Li’s article (contribution 10) investigated the potential associations between PFASs and polycystic ovary syndrome. They identified profiles of PFASs in follicular fluid and suggested that PFOA may be a risk factor for PCOS. Li et al.’s study provides important evidence for preventing reproductive health damage in relation to the persistence and accumulation of PFASs.

## 3. Conclusions

In essence, these 10 comprehensive studies significantly contribute to our understanding of the intricate connections between environmental exposure and reproductive health. Through employing diverse methodologies, including cellular assays, clinical investigations, and animal models, their findings underscore the importance of addressing environmental exposure in order to protect reproductive health.

We extend our heartfelt gratitude to the authors, reviewers, and editorial team for their invaluable contributions to this Special Issue. Your dedication and expertise have been instrumental in shaping this exploration of environmental exposure and reproductive health.

## References

[1] Segal T., Giudice L. (2019). Before the beginning: Environmental exposures and reproductive and obstetrical outcomes. Fertil. Steril..

[2] Sciorio R., Tramontano L., Adel M., Fleming S. (2024). Decrease in Sperm Parameters in the 21st Century: Obesity, Lifestyle, or Environmental Factors? An Updated Narrative Review. J. Pers. Med..

[3] Toppari J., Larsen J., Christiansen P., Giwercman A., Grandjean P., Guillette L., Jégou B., Jensen T., Jouannet P., Keiding N. (1996). Male reproductive health and environmental xenoestrogens. Environ. Health Perspect..

[4] Liu Y., Gong S., Ye L., Li J., Liu C., Chen D., Fang M., Letcher R., Su G. (2021). Organophosphate (OP) diesters and a review of sources, chemical properties, environmental occurrence, adverse effects, and future directions. Environ. Int..

[5] Zhang D., Lu S. (2023). A holistic review on triclosan and triclocarban exposure: Epidemiological outcomes, antibiotic resistance, and health risk assessment. Sci. Total Environ..

[6] Wu M., Dong W., Guan D., Li S., Ma L. (2024). Total contents, fractionation and bioaccessibility of nine heavy metals in household dust from 14 cities in China. Environ. Res..

